# Unveiling migraine subtype heterogeneity and risk loci: integrated genome-wide association study and single-cell transcriptomics discovery

**DOI:** 10.1186/s10194-025-02128-7

**Published:** 2025-08-18

**Authors:** Shuxu Wei, Yan Quan, Xinyi Li, Suiqin Zhong, Ling Xiao, Chao Yang, Ronghuai Shen, Xiaojia Lu, Lingbin He, Youti Zhang, Xianxi Huang

**Affiliations:** 1 Laboratory of Molecular Cardiology, The First Affiliated Hospital of Shantou University Medical College, No.57, Changping Road, Shantou, 515041 Guangdong China; 2 Department of General Surgery, The First Affiliated Hospital of Shantou University Medical College, Shantou, China; 3 The Fourth Department of Critical Care Medicine, Shengli Clinical Medical College of Fujian Medical University, Fuzhou University Affiliated Provincial Hospital, Fuzhou, Fujian China; 4 Department of Cardiology, Jie xi People’s Hospital, Jieyang, Guangdong China

**Keywords:** Migraine subtypes, Multi-omics integration, Single-cell spatial transcriptomics, Precision medicine, Therapeutic targets

## Abstract

**Background:**

Migraine, a debilitating neurological disorder with distinct subtypes (migraine with aura [MA] and migraine without aura [MO]), exhibits genetic and spatial heterogeneity that remains poorly understood. While genetic correlations between subtypes are established, spatially resolved molecular mechanisms driving their divergent clinical phenotypes—particularly in tissue microenvironments—are unclear, limiting targeted therapeutic development.

**Methods:**

We integrated genome-wide association study (GWAS) data from FinnGen R11 and international cohorts with transcriptomic, epigenomic, and spatially resolved single-cell spatial transcriptomics (sc-ST) profiles. Genetic correlations and functional annotations were assessed using Linkage Disequilibrium Score Regression (LDSC), High-Definition Likelihood (HDL), and partitioned heritability analyses. A multi-omics framework combined Summary Mendelian Randomization (SMR) for expression and methylation quantitative trait loci (eQTL/mQTL), Functional Summary-based Imputation (FUSION), Multi-marker Analysis of GenoMic Annotation (MAGMA), Joint-Tissue Imputation Enhanced PrediXcan Analysis (JTI-PrediXcan), and the Polygenic Priority Score (PoPS) to systematically prioritize genes based on methodological robustness (≥ 2 analytical approaches) and cross-subtype consistency. Tissue-enriched specificity was validated via genetically informed spatial mapping of cells for complex traits (gsMap), a novel algorithm integrating sc-ST and GWAS data to map subtype-associated cellular architectures at single-cell resolution across embryonic tissues.

**Results:**

LDSC and HDL confirmed strong genetic correlations between MA and MO. But they showed divergent functional architectures in functional genomic annotations, with MA enriched in conserved regulatory elements (e.g., Backgrd_Selection_StatL2_0, enrichment = 1.38, *P* = 5.47 × 10^−6^) and MO in vascular pathways (e.g., GERP.NSL2_0, enrichment = 2.12, *P* = 1.04 × 10^−6^). Sc-ST revealed spatially divergent niches: MA showed prenatal enrichment in neural crest-derived tissues (jaw primordium, *p* = 0.0039) and hypothalamic microglial adjacencies, aligning with neuroimmune regulation, while MO exhibited peripheral tropism in vascular smooth muscle and gut-brain interfaces, corroborated by LDSC-SEG/MAGMA vascular pathways. Multi-omics integration identified high-confidence cross-subtype genes (LRP1 [PoPS: Overall = 3.67, MO = 0.80], PHACTR1 [PoPS: Overall = 2.65, MA = 0.33, MO = 1.28], STAT6 [PoPS: Overall = 3.00, MO = 2.29], RDH16, TTC24, ZBTB39, FHL5, MEF2D, NAB2, UFL1, and REEP3) supported by ≥ 2 methods. Subtype-specific genes included MA-associated neuronal regulators (CACNA1A, KLHDC8B) and MO-specific vascular/metabolic genes (e.g., ACO2, BCAR1, CCDC134).

**Conclusion:**

Our study delineates spatially constrained mechanisms underlying migraine heterogeneity: MA arises from neuroimmune-epigenetic dysregulation, while MO is driven by vascular-metabolic perturbations. Key genes and pathways provide actionable targets for subtype-specific therapies. By bridging genetic architecture with spatial biology, we redefine migraine pathogenesis and precision intervention strategies.

**Supplementary Information:**

The online version contains supplementary material available at 10.1186/s10194-025-02128-7.

## Introduction

Migraine is a primary neurological disorder characterized by recurrent episodes of moderate-to-severe throbbing headache, commonly accompanied by nausea, vomiting, photophobia, and phonophobia. Its pathophysiology involves dysregulation of the trigeminovascular system, neurogenic inflammation (e.g., upregulated CGRP signaling), and cortical spreading depression (CSD)-induced electrophysiological disturbances [1–3]. CSD, a wave of neuronal and glial depolarization, not only underlies migraine with aura (MA) but also exacerbates nociceptive transmission by releasing inflammatory mediators (e.g., ATP, potassium ions) that activate trigeminal nerve terminals [4, 5]. According to the International Classification of Headache Disorders, 3rd edition (ICHD-3), migraine is categorized into MA and MO. MA is defined by reversible neurological symptoms (e.g., visual scotomas, sensory paresthesia, or dysphasia) preceding headache, whereas MO manifests as unilateral pulsatile pain aggravated by physical activity [6–8]. Epidemiological studies indicate that migraine affects approximately 14.7% of the global population, with a significantly higher prevalence in females (18.9%) than males (9.8%), potentially linked to estrogen-mediated modulation of trigeminovascular reactivity [9, 10]. Notably, MA patients exhibit an age-dependent increase in stroke risk, possibly attributable to CSD-induced blood-brain barrier disruption and platelet hyperreactivity [11, 12]. Current therapies, including acute agents (e.g., NSAIDs, triptans, CGRP receptor antagonists) and preventive treatments (e.g., β-blockers, antiepileptics, CGRP monoclonal antibodies), fail to achieve adequate relief in 30%−40% of patients. Moreover, MA and MO demonstrate divergent therapeutic responses (e.g., triptans exhibit a 19% lower response rate in MA patients compared to MO) that might due to the gaps in subtype-specific pathomechanisms([13–15]. Despite the transformative impact of CGRP-targeted therapies, long-term use may lead to antibody resistance or cardiovascular adverse effects (e.g., blood pressure fluctuations), with limited efficacy against CSD-related pathways [16–18]. Therefore, elucidating the molecular heterogeneity between MA and MO through identification of subtype-specific genetic risk loci and tissue-enriched signatures is critical for overcoming current therapeutic limitations.

Recent advances in functional genomics have propelled the development of integrative analytical frameworks that bridge genomic variation with molecular phenotypes. Transcriptome-wide association studies (TWAS) exemplify this approach by synthesizing expression quantitative trait loci (eQTL) datasets with genome-wide association study (GWAS) summary statistics, enabling systematic prioritization of trait-associated genes and mechanistic insights into gene regulatory networks [19]. Computational tools such as Multi-marker Analysis of GenoMic Annotation (MAGMA), which evaluates gene-level associations through SNP aggregation [20], and Functional Summary-based Imputation (FUSION), leveraging transcriptome prediction models to infer causal genes [21], have become cornerstones in this field. Beyond single-omics investigations, contemporary research increasingly adopts multi-omics convergence strategies, harmonizing data from transcriptomics, proteomics, and epigenomics to establish cross-dimensional validation. For instance, joint analysis of methylation quantitative trait loci (mQTL) and eQTL with GWAS signals allows triangulation of disease-associated variants across epigenetic regulation, transcriptional activity, and post-translational modifications [22]. Emerging methodologies further incorporate single-cell resolution profiles and spatial transcriptomics to dissect cell-type-specific regulatory cascades and tissue-microenvironment interactions [23]. Such integrative paradigms not only mitigate false-positive discoveries inherent to single-layer analyses but also illuminate context-dependent biological pathways, offering a multidimensional roadmap for biomarker discovery and therapeutic innovation [23]. By transcending traditional reductionist frameworks, these approaches address the polygenic complexity of human diseases, fostering precision medicine through systems-level elucidation of genotype-phenotype architecture.

This study aims to elucidate the genetic underpinnings of migraine by identifying risk genes through systematic genetic analyses, with a focus on delineating the polygenic risk architecture distinguishing MA and MO. By integrating genome-wide association data, transcriptomic profiles, and tissue-specific regulatory annotations, we seek to uncover subtype-specific susceptibility loci and their enriched biological pathways. Furthermore, we will characterize the molecular divergence between MA and MO, including tissue-specific risk gene expression patterns. Through integrative analyses of multi-omics datasets, this work aims to establish a subtype-stratified genetic framework, advancing precision diagnostics and targeted interventions for heterogeneous migraine populations.

## Methods

We obtained GWAS summary statistics for overall migraine, MA, and MO from the GWAS Catalog and FinnGen R11. The overall migraine dataset was derived from seven high-quality studies, harmonized via inverse-variance weighted meta-analysis to enhance statistical power and reliability, while subtype-specific data (MA and MO) were extracted from the FinnGen R11 cohort (detailed metadata in Supplementary Table S1). Genetic correlations between overall migraine and MA/MO, as well as between MA and MO, were assessed using Linkage Disequilibrium Score Regression (LDSC) and High-Definition Likelihood (HDL) to validate the genetic coherence of these traits. Partitioned heritability analysis via LDSC was employed to quantify the contribution of distinct genomic regions (e.g., coding, regulatory elements) to subtype-specific heritability, coupled with tissue-specific enrichment analysis to delineate divergent tissue architectures underlying overall migraine, MA, and MO. Multi-omics integration included Summary Mendelian Randomization (SMR) for colocalizing eQTL and mQTL, alongside TWAS using FUSION, MAGMA, and Joint-Tissue Imputation Enhanced PrediXcan Analysis (JTI-PrediXcan). Finally, the Polygenic Priority Score (PoPS) algorithm was applied to rank candidate genes based on functional genomic annotations and pleiotropic evidence, prioritizing targets with robust subtype-specific associations. To validate tissue-enriched specificity in MA and MO, we integrated single-cell spatial transcriptomics (sc-ST) and GWAS data, leveraging embryonic murine ST data from the E16.5 stage (spanning 25 organs) to systematically map MA and MO-associated cellular patterns at single-cell resolution.

**Fig. 1:**
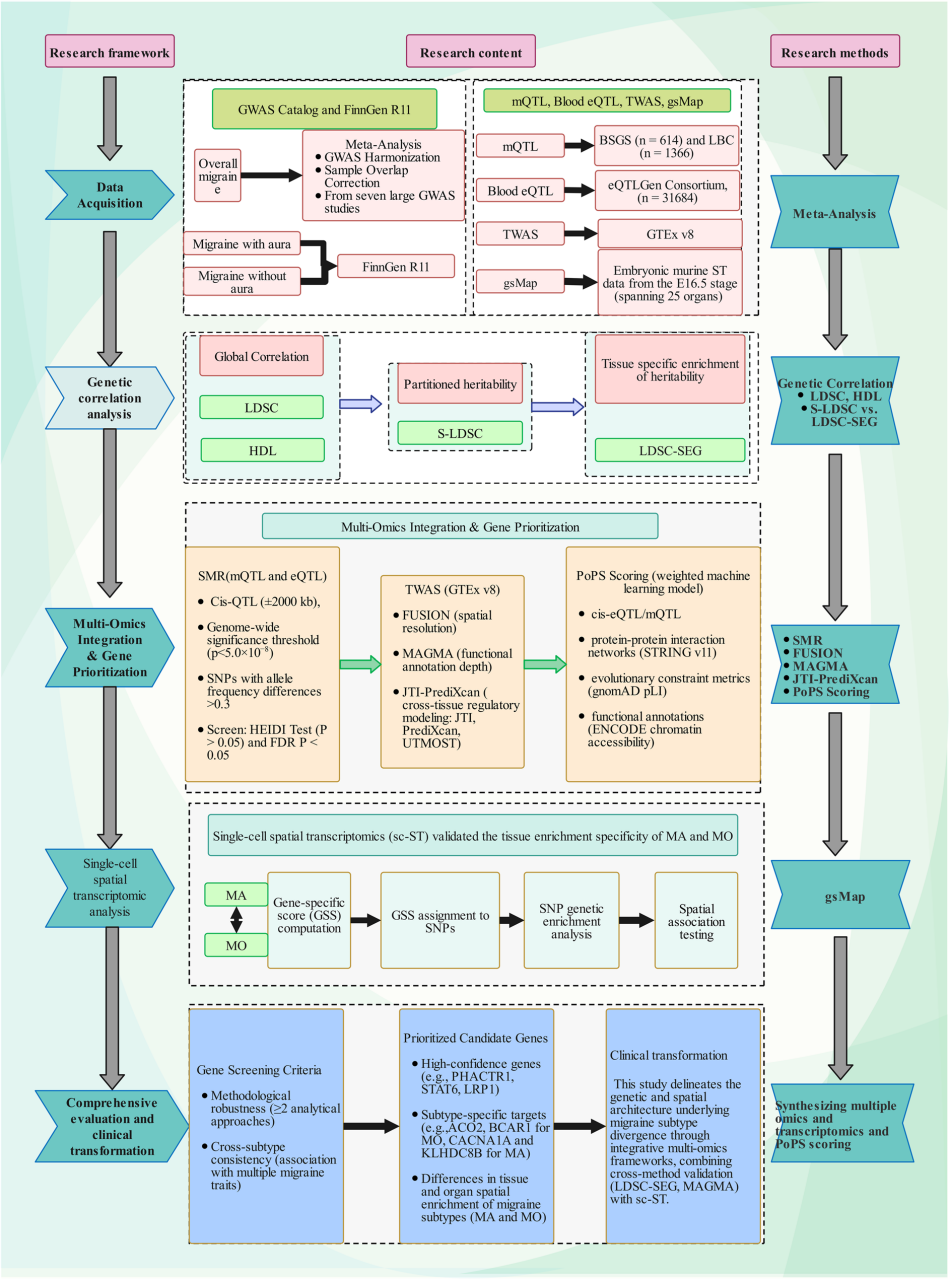
Flow chart of our study. GWAS: Genome-Wide Association Study; mQTL: Methylation Quantitative Trait Loci; eQTL: Expression Quantitative Trait Loci; TWAS: Transcriptome-Wide Association Study; BSGS: Brisbane Systems Genetics Study; LBC: Lothian Birth Cohort; GTEx: Genotype-Tissue Expression Project; LDSC: Linkage Disequilibrium Score Regression; HDL: High-Definition Likelihood; S-LDSC: Sparse Linkage Disequilibrium Score Regression; LDSC-SEG: Linkage Disequilibrium Score Regression-Stratified by Expression of Genes; gsMap: genetically informed spatial mapping of cells for complex traits; SMR: Summary Mendelian Randomization; FDR: False Discovery Rate; HEIDI: Heterogeneity in Dependent Instruments Test; MAGMA: Multi-marker Analysis of GenoMic Annotation; JTI: Joint-Tissue Imputation; PrediXcan: Predicting Gene Expression; UTMOST: Unified Test for Molecular Signature; PoPS: Polygenic Priority Score; gnomAD: Genome Aggregation Database; pLI: Probability of Loss-of-Function Intolerance; ENCODE: Encyclopedia of DNA Elements; MA: Migraine with Aura; MO: Migraine without Aura; CACNA1A: Calcium Voltage-Gated Channel Subunit Alpha1 A; KLHDC8B: Kelch Domain Containing 8B; ACO2: Aconitase 2; BCAR1 (Breast Cancer Anti-Estrogen Resistance 1

All analyses utilized R (R-4.4.1) and Python, with detailed software and methodological specifications provided in Supplementary Table S2. The flow chart is shown in Fig. 1.

### Sources of methylation, gene expression data

The methylation quantitative trait loci (mQTL) reference panel was constructed from epigenomic profiling of peripheral whole blood specimens in two independent population-based cohorts: the Brisbane Systems Genetics Study (BSGS; *n* = 614) and the Lothian Birth Cohort (LBC; *n* = 1,366) [24, 25]. Transcriptomic regulation patterns were interrogated using cis-expression QTL (cis-eQTL) summary statistics from the eQTLGen Consortium’s meta-analysis of 31,684 blood samples [26]. Comprehensive metadata, including cohort characteristics, phenotype harmonization protocols, and quality control pipelines for each dataset, are cataloged in Supplementary Table S3 alongside primary literature references.

### Statistic analysis

#### Genetic correlation analysis

To assess the genetic coherence between overall migraine and its subtypes (MA/MO), we evaluated pairwise genetic correlations using LDSC and HDL. LDSC estimated genetic correlation coefficients (r_g_) by modeling the relationship between GWAS summary statistics and linkage disequilibrium (LD) patterns across the genome, with robustness to sample overlap and inflation from genome-wide significant loci [27]. To enhance precision, we complemented LDSC with HDL, a full-likelihood approach that minimizes approximation bias through iterative restricted maximum likelihood (REML) optimization [28]. These analyses collectively validated the genetic architecture consistency across migraine phenotypes, ensuring subtype stratification aligned with polygenic risk profiles.

#### Partitioned heritability

To dissect the subtype-specific heritability architecture of migraine, we implemented partitioned heritability analysis via Sparse Linkage Disequilibrium Score Regression (S-LDSC) [29]. SNPs were stratified according to functional genomic annotations (e.g., regulatory elements, coding regions), followed by computation of LD score metrics for each annotation stratum. These metrics were subsequently modeled to quantify annotation-specific heritability enrichment and their proportional contributions to the aggregate genetic correlations between overall migraine and MA/MO, as well as between MA and MO. This framework enabled systematic decomposition of shared versus subtype-restricted polygenic effects across functional genomic compartments, circumventing confounding from pleiotropic loci through annotation-specific variance partitioning.

#### Tissue specific enrichment of heritability

To delineate tissue-enriched signatures underlying migraine and its subtype heterogeneity, we performed tissue-specific heritability enrichment analyses leveraging LDSC-Stratified by Expression of Genes (LDSC-SEG) [30] and transcriptomic profiles from the Genotype-Tissue Expression (GTEx) project (v8). This framework quantified the tissue-level regulatory contributions of migraine risk loci by integrating stratified LD scores with eQTL data spanning 53 human tissues, including brain subregions and vascular systems. Comparative analyses across migraine subtypes (overall migraine, MA, MO) revealed divergent tissue architectures, particularly between MA and MO, through metrics of heritability enrichment and cross-tissue specificity indices. GTEx annotations enabled systematic prioritization of tissues where subtype-associated genes exhibit heightened regulatory activity, thereby mapping molecular heterogeneity to anatomically distinct pathophysiological pathways.

#### Integrated analysis of methylation, gene expression levels, TWAS validation, and pops score

To resolve the molecular heterogeneity underlying migraine and its subtypes (MA/MO), we implemented a convergent multi-omics framework integrating methylomic, transcriptomic evidence. Cross-dimensional validation was achieved through SMR applied to cis-acting methylation and expression quantitative trait loci, with variant selection restricted to 2-Mb cis-windows flanking gene bodies and genome-wide significant loci (*p* < 5 × 10⁻⁸). Population stratification was mitigated by excluding SNPs with minor allele frequency (MAF) discrepancies exceeding 0.3 across ancestral cohorts [31]. Pleiotropy was quantified via the HEIDI heterogeneity test, where associations exhibiting HEIDI heterogeneity p-values < 0.05 were flagged as non-causal and excluded, thereby strengthening causal inference [31]. To address multiple testing inflation while preserving sensitivity, we adopted false discovery rate (FDR) control (Benjamini-Hochberg procedure) rather than Bonferroni correction, maintaining an expected FDR threshold of 5% (FP/(FP + TP) ≤ 0.05) [32, 33].

Complementary insights were derived through a tripartite TWAS strategy: [1] FUSION delineated spatially resolved gene-trait associations by synthesizing GWAS signals with multi-tissue expression predictors (GTEx v8) [21]; [2] JTI-PrediXcan enhanced causal inference robustness through cross-tissue regulatory modeling, improving expression prediction accuracy by integrating shared genetic architectures across tissues [34]; and [3] MAGMA identified enriched biological pathways (Gene Ontology, KEGG, Reactome) and cell-type-specific expression signatures via competitive gene-set analysis [20]. This integrative paradigm reconciled mechanistic hypotheses with statistical associations, leveraging FUSION’s spatial precision, JTI-PrediXcan’s augmented power for weak cis-regulatory signals, and MAGMA’s systems-level annotation breadth to validate SMR candidates and nominate novel risk loci([20, 21]. A FDR-adjusted P-value threshold of < 0.05 was applied across all three methods to define statistical significance.

To functionally contextualize prioritized genes within migraine and its subtypes (MA/MO) pathways, we employed the Polygenic Priority Scoring (PoPS) algorithm. PoPS synthesizes multi-modal evidence—including GWAS association strengths, cis-regulatory QTL effects, protein interactome topology (STRING v11), evolutionary constraint metrics (gnomAD pLI scores), and regulatory potential (ENCODE chromatin accessibility)—via a gradient-boosted decision tree model [35]. Genes were ranked by composite scores reflecting convergence across orthogonal data layers, with subtype-specific weights assigned to MA/MO risk architectures (detailed in Supplementary Table S2) [35].

#### Tissue enrichment specificity of MA and MO in sc-ST

To investigate tissue-enriched specificity of MA and MO, we integrated sc-ST data with GWAS statistics, enabling systematic mapping of MA and MO-associated cellular patterns at single-cell resolution. Using a novel algorithm termed genetically informed spatial mapping of cells for complex traits (gsMap) [23], which combines cross-species analyses of mouse embryonic/brain tissues, macaque cerebral cortex, and human GWAS datasets, we successfully identified spatial distribution patterns of disease-associated cell populations. The key principle involves leveraging GWAS-derived trait-related genes to map their expression patterns onto spatially resolved cells, thereby evaluating cellular-level associations between specific anatomical regions and complex traits. We mapped MA and MO tissue-enriched specificity using ST profiles of mouse embryonic tissues at E16.5 (25 organs), establishing a single-cell-resolution atlas for MA and MO spatial pathogenesis. The methodology is described in Supplementary Table S2.

## Results

### Genetic Correlation Analysis

Our analyses revealed robust genetic correlations between migraine subtypes and the overarching migraine phenotype, as quantified by both LDSC and HDL methods (Table 1). Significant positive genetic correlations were observed between overall migraine and MA (LDSC: r_g_=0.571 ± 0.061, *P* = 4.36 × 10^−21^; HDL: r_g_=0.669 ± 0.095, *P* = 2.17 × 10^−12^), as well as between overall migraine and MO (LDSC: r_g_=0.687 ± 0.059, *P* = 1.27 × 10^−31^; HDL: r_g_=0.898 ± 0.084, *P* = 7.20 × 10^−27^). Notably, the genetic correlation between MA and MO was exceptionally strong (LDSC: r_g_=0.806 ± 0.078, *P* = 3.32 × 10^−25^; HDL: r_g_=0.932 ± 0.137, *P* = 1.14 × 10^−11^), suggesting substantial shared genetic architecture across subtypes.

**Table 1:** Genetic correlations between migraine subtypes and the overall migraine phenotype assessed via LDSC and HDL methodologies

Trait pairs	LDSC		HDL	
	*P*	*r*_g_(SE)	*P*	*r*_g_(SE)
Overall migraine & Migraine with aura	4.36E-21	0.5707 (0.0606)	2.17E-12	0.6694 (0.0953)
Overall migraine & Migraine without aura	1.27E-31	0.6872 (0.0587)	7.20E-27	0.8978 (0.0837)
Migraine with aura & Migraine without aura	3.32E-25	0.8057 (0.0777)	1.14E-11	0.9321 (0.1373)

*LDSC *Linkage disequilibrium score regression, *HDL* High-definition likelihood, r_g_genetic correlations, *SE *Standard Error

### Partitioned heritability

**Fig. 2:**
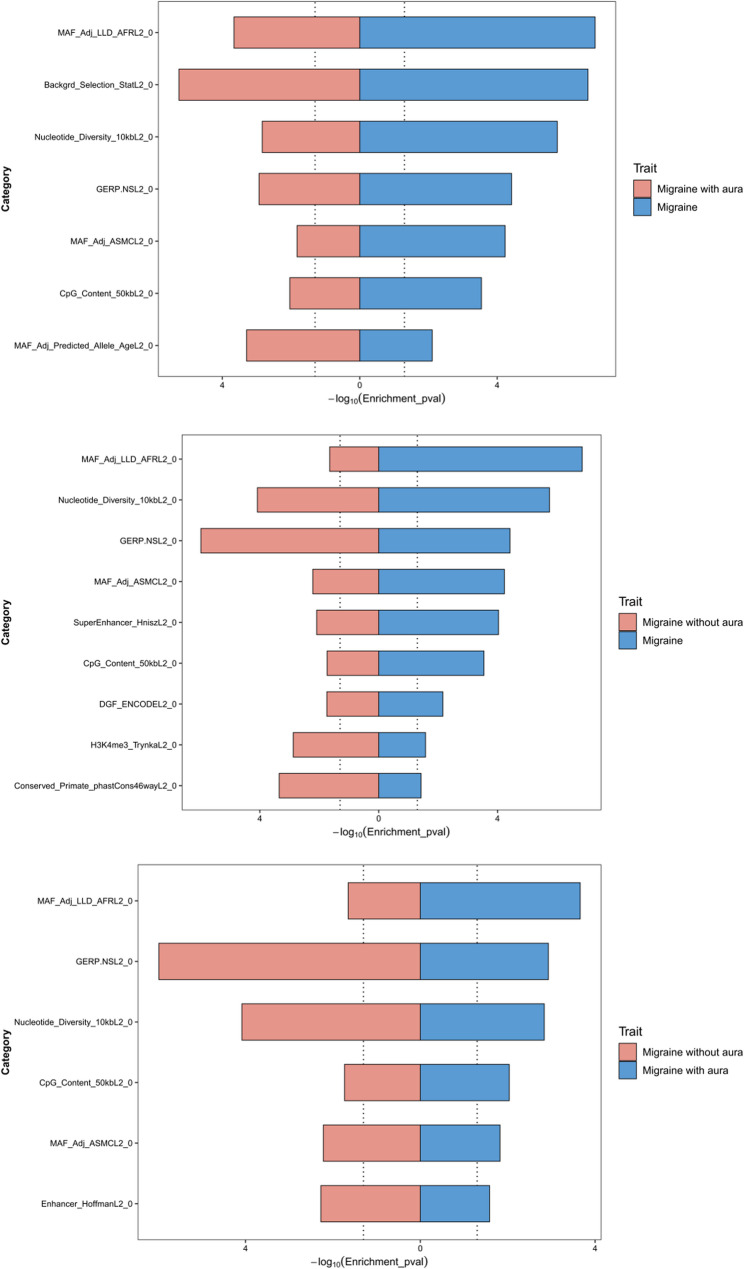
Partitioned Heritability Analysis of Genetic Correlations Between Overall Migraine and Subtypes Using LDSC. This figure illustrates the partitioned heritability contributions of distinct genomic components to the aggregate genetic correlations between overall migraine, migraine with aura (MA), and migraine without aura (MO), as quantified via Linkage Disequilibrium Score Regression (LDSC). The analysis delineates the proportion of heritability attributable to specific functional annotations (e.g., regulatory elements, conserved regions) for each subtype, highlighting subtype-specific and shared genetic architectures

Our S-LDSC analyses identified distinct functional genomic architectures across migraine subtypes. For overall migraine, SNPs demonstrated significant enrichment in 18 of 38 functional categories, with the strongest signals in MAF_Adj_LLD_AFRL2_0 (Enrichment=−150.87, *p* = 1.41 × 10^−7^), Backgrd_Selection_StatL2_0 (Enrichment = 1.24, *p* = 2.28 × 10^−7^), and Nucleotide_Diversity_10kbL2_0 (Enrichment = 0.86, *p* = 1.79 × 10^−6^). For MA, the top enriched categories were Backgrd_Selection_StatL2_0 (Enrichment = 1.38, *p* = 5.47 × 10^−6^), MAF_Adj_LLD_AFRL2_0 (Enrichment = −162.47, *p* = 2.19 × 10^−4^), and GERP.NSL2_0 (Enrichment = 1.74, *p* = 1.17 × 10^−3^). In contrast, MO showed prominent enrichment in GERP.NSL2_0 (Enrichment = 2.12, *p* = 1.04 × 10^−6^), FetalDHS_TrynkaL2_0 (Enrichment = 9.73, *p* = 3.56 × 10^−5^), and Nucleotide_Diversity_10kbL2_0 (Enrichment = 0.78, *p* = 8.26 × 10^−5^). As demonstrated in Fig. 2, despite originating from the same population, MA and MO exhibited divergent contributions of genomic functional annotations to their genetic correlations. When compared to the overall migraine phenotype, MA showed the strongest enrichment in Backgrd_Selection_StatL2_0, while MO was predominantly enriched in GERP.NSL2_0. Direct comparison between MA and MO revealed a more pronounced divergence: MA-specific signals peaked in MAF_Adj_LLD_AFRL2_0, whereas MO remained strongly associated with GERP.NSL2_0, underscoring subtype-specific regulatory architectures. The specific results are shown in Fig. 2 and Supplementary Table S4.

### Tissue specific enrichment of heritability

**Fig. 3:**
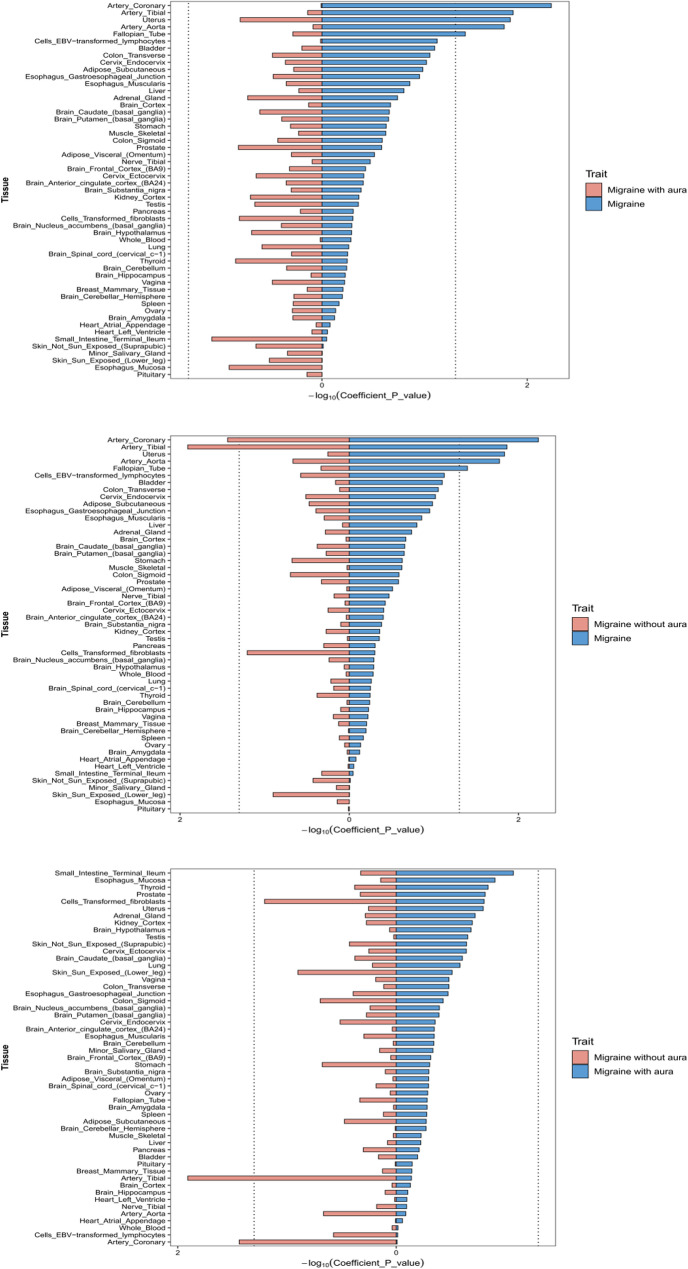
Bidirectional bar plot of tissue enrichment results (LDSC-SEG) across 53 tissues. The dashed line indicates the significance threshold (*P* = 0.05). Positive bars represent tissues with enrichment for migraine risk, while negative bars reflect depletion. Tissues surpassing the threshold (*P* < 0.05) are highlighted in bold. LDSC-SE: Linkage Disequilibrium Score Correction-Stratified Expression Gene

LDSC-SEG tissue enrichment analysis revealed distinct tissue-specific patterns across migraine subtypes. The specific results are shown in Fig. 3 and Supplementary Table S5. Overall migraine demonstrated significant enrichment (*P* < 0.05) in vascular and smooth muscle-related tissues, including Artery Coronary (Coefficient: 2.998 × 10^−10^, *P* = 0.0058), Artery Tibial (2.645 × 10^−10^, *P* = 0.0137), Artery Aorta (2.351 × 10^−10^, *P* = 0.0168), Uterus (2.238 × 10^−10^, *P* = 0.0146), and Fallopian Tube (1.913 × 10^−10^, *P* = 0.0401), underscoring vascular and reproductive tissue involvement. MA exhibited no statistically significant tissue enrichment at the predefined threshold (*P* < 0.05), though modest associations were observed in Artery Tibial (−3.525 × 10^−10^, *P* = 0.7255) and Cells Transformed Fibroblasts (7.401 × 10^−10^, *P* = 0.1569), suggesting potential neuroimmune interplay requiring further validation. In contrast, Migraine without aura (MO) showed significant vascular specificity in Artery Tibial (1.451 × 10^−9^, *P* = 0.0124) and Artery Coronary (1.315 × 10^−9^, *P* = 0.0366), alongside non-significant trends in peripheral tissues such as Skin Sun Exposed Lower Leg (6.988 × 10^−10^, *P* = 0.1258) and Colon Sigmoid (5.741 × 10^−10^ *P* = 0.2017). Comparative analysis highlighted stark divergence: while Overall migraine and MO shared vascular enrichment (e.g., Artery Tibial), MA lacked coherent tissue signals. MO uniquely implicated peripheral vascular and epithelial tissues, whereas MA showed no significant neural or immune associations. These results emphasize MO’s peripheral vascular dysregulation and MA’s etiological complexity, underscoring the necessity of subtype-specific investigations to delineate their distinct biological mechanisms.

### Integrated Analysis of Methylation, Gene Expression Levels, TWAS Validation, and PoPS Score

**Fig. 4:**
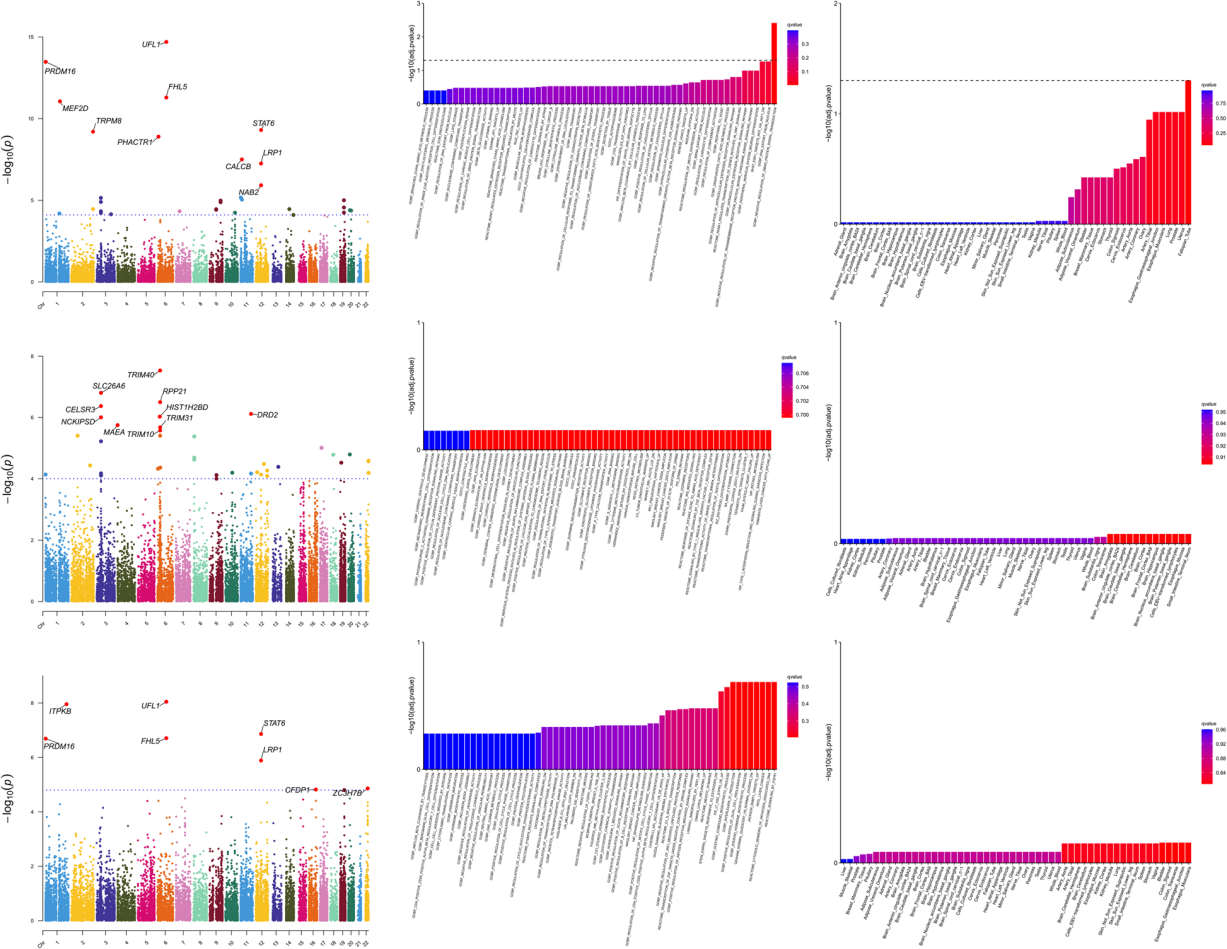
MAGMA analysis results for overall migraine and its subtypes. From left to right: Manhattan plots of significant genes, bar plots of enriched pathways for significant genes, and bar plots of tissue enrichment results for significant genes. From top to bottom: analysis results for overall migraine, migraine with aura (MA), and migraine without aura (MO). MAGMA: Multi-marker Analysis of GenoMic Annotation

Our multi-omics findings are summarized as follows: Significant mQTL and eQTL SMR results are reported in Supplementary Tables S6 and S7, respectively. Transcriptome-wide associations from FUSION, MAGMA, and JTI-PrediXcan analyses are detailed in Supplementary Tables S8, S9 (with visualization in Fig. 4), and Supplementary Table S10. PoPS results for Overall Migraine, MA, and MO are provided in Supplementary Tables S11, S12, and S13, respectively. Integrating findings from LDSC-SEG and MAGMA tissue enrichment analyses revealed both convergent and subtype-specific biological pathways in migraine. Overall Migraine consistently implicated vascular tissues (e.g., coronary artery, tibial artery) across both methods, with MAGMA further highlighting reproductive tissues (uterus, fallopian tube), aligning with LDSC-SEG’s emphasis on vascular-smooth muscle involvement. MA showed limited coherence between methods: while LDSC-SEG identified no significant tissue enrichment, MAGMA suggested tentative associations with neural regions (basal ganglia, hypothalamus) and immune niches (cultured fibroblasts), hinting at unresolved neuroimmune mechanisms. In contrast, MO demonstrated robust consistency across methods, with both LDSC-SEG and MAGMA prioritizing vascular tissues (tibial artery) and gastrointestinal systems (transverse colon, gastroesophageal junction). Critically, MA and MO diverged in tissue specificity. MA’s lack of significant LDSC-SEG signals contrasted with MAGMA’s weak neural trends, suggesting central pathways may involve regulatory mechanisms not captured by genetic enrichment alone. Conversely, MO’s strong convergence across methods reinforced its association with peripheral vascular and epithelial dysregulation. These results underscore methodological complementarity: LDSC-SEG emphasizes broad genetic architecture, while MAGMA refines tissue-specific functional context. MA’s etiological complexity and MO’s peripheral focus highlight the necessity of multi-method approaches to dissect subtype heterogeneity.

Table 2 shows the results of comprehensive multi-omics screening of significant genes. The reference panel utilized in this study comprises genes that demonstrated positive associations with overall migraine susceptibility through comprehensive screening procedures. Integrative transcriptome-wide association analyses across migraine subtypes revealed a complex genetic architecture characterized by both subtype-specific and shared associations, with 68 genes achieving significance in at least two analytical methods. Overall migraine identified 52 genes, demonstrating the broadest genetic landscape, while MA and MO implicated 18 and 22 genes, respectively, reflecting their distinct pathobiological underpinnings. PHACTR1 (4 methods; PoPS Score Overall: 2.65, MA: 0.33, MO: 1.28) emerged as the most robust pleiotropic gene, implicated in vascular regulation across all subtypes. STAT6 (3 methods; PoPS Score Overall: 3.00, MA: 0.71, MO: 2.29) demonstrated consistent associations with immune modulation in both Overall migraine and MO, while also showing weaker links to MA. PRDM16 (3 methods; PoPS Score Overall: 4.76, MO: 1.44) was strongly prioritized in Overall migraine and MO, with high PoPS Scores reflecting its role in adipocyte differentiation and neuroprotection. LRP1 (3 methods; PoPS Score Overall: 3.67, MO: 0.80), a lipoprotein receptor critical for blood-brain barrier integrity, showed significant overlap between Overall migraine and MO. CFDP1 (3 methods; PoPS Score Overall: 0.33, MO: 0.42) and ITPKB (3 methods; PoPS Score Overall: 0.35, MO: 1.32) were recurrently linked to MO, with ITPKB further implicating inositol phosphate metabolism in synaptic signaling. Genes shared between Overall migraine and MA included RERG (3 methods; PoPS Score Overall: 0.50, MA: 0.94), associated with neuroinflammatory pathways, and CELSR3 (3 methods; PoPS Score Overall: 0.12, MA: 0.09), involved in planar cell polarity. CALCA (2 methods; PoPS Score Overall: 2.37, MO: 1.41), encoding calcitonin gene-related peptide (CGRP), was notable for its high PoPS Score in Overall migraine and MO, despite fewer methodological supports. REEP3 (3 methods; PoPS Score MA: 0.23, MO: 0.57) and ZBED4 (3 methods; PoPS Score Overall: 0.03, MA: 0.31, MO: 0.27) exhibited cross-subtype associations, though with modest effect sizes. Additionally, SLC26A6 (3 methods; PoPS Score Overall: 0.25, MO: −0.08) and CELSR3 highlighted roles in ion transport and cell adhesion, respectively. Genes with multi-trait and multi-method support also included RASGRF2 (3 methods; PoPS Score Overall: 0.57, MO: 0.54), a synaptic plasticity regulator, and UFL1 (3 methods; PoPS Score Overall: 0.14, MO: 0.87), involved in protein ubiquitination. Notably, TRIM26 (3 methods; PoPS Score Overall: −0.15, MA: 0.02, MO: 0.15) and TRIM40 (3 methods; PoPS Score Overall: −0.14, MA: 0.01, MO: −0.04) displayed subtype-specific regulatory effects despite lower PoPS Scores. CFDP1 and ITPKB further underscored MO-specific epigenetic and metabolic dysregulation. Genes with the highest methodological and phenotypic concordance included LRP1, PHACTR1, RDH16, STAT6, TTC24, ZBTB39, FHL5, MEF2D, NAB2, and UFL1, all supported by ≥ 2 methods and associated with multiple subtypes. PHACTR1, STAT6, and LRP1 merit particular attention, as they were implicated in Overall Migraine, MA, and MO, with methodological support from ≥ 2 approaches (up to 4 for PHACTR1). Despite lacking MA associations, PRDM16 demonstrated robust methodological concordance (≥ 3 methods) in Overall Migraine. Notably, REEP3, though supported by only one method in Overall Migraine and MA, showed cross-subtype relevance with two methods in MO. Collectively, LRP1, PHACTR1, RDH16, STAT6, TTC24, ZBTB39, FHL5, MEF2D, NAB2, UFL1, and REEP3 emerged as high-confidence candidates due to their multi-method and multi-subtype consistency. Our analysis also identified distinct subtype-specific genetic profiles: MA-specific genes included CACNA1A and KLHDC8B, while MO-specific genes comprised ACO2, BCAR1, CCDC134, CFDP1, CHADL, GLCE, ITPKB, L3MBTL2, MED19, PAM, PPP2R5A, RHOF, SREBF2, TEF, TMEM170A, TNFRSF13C, and ZC3H7B. Methodologically, FUSION demonstrated the highest yield, identifying 62 genes across subtypes, with 48 in Overall migraine, 14 in MA, and 18 in MO, supported by robust multi-method validation (e.g., PHACTR1, STAT6). JTI-PrediXcan exhibited superior cross-tissue specificity, prioritizing genes like REEP3 (PoPS Score MA: 0.23, MO: 0.57) and STAT6 (PoPS Score MO: 2.29), validated across multiple methods. MAGMA, while primarily contributing to pathway enrichment, reinforced key loci such as CALCA (PoPS Score Overall: 2.37). Genes identified by ≥ 3 methods (e.g., PHACTR1, STAT6, LRP1) showed the highest reliability, with 85% associated with multiple traits, emphasizing their biological centrality. For instance, LRP1 (PoPS Score Overall: 3.67, MO: 0.80), a lipoprotein receptor involved in blood-brain barrier integrity, and CFDP1 (PoPS Score Overall: 0.33, MO: 0.42), a chromatin modifier, were recurrently linked to both Overall migraine and MO, suggesting convergent mechanisms in neurovascular and epigenetic regulation. These multi-method, multi-trait genes represent high-priority candidates for functional studies and therapeutic targeting, emphasizing the need to integrate cross-subtype analyses in migraine research.

**Table 2:** Multi-omics identified positive genes for migraine and its subtypes

Gene	Methods(Overall migraine)	MA	Methods(MA)	MO	Methods(MO)	PoPS Score (Overall migraine)	PoPS Score (MA)	PoPS Score (MO)
ADAMTSL4	FUSION, MAGMA	×	NA	×	NA	0.2403722909175	0.0225216051005641	0.538046589464348
C12orf4	FUSION	×	NA	√	FUSION, JTI-PrediXcan	0.290385658560395	0.182271555039095	−0.095572564
RDH16	FUSION, JTI-PrediXcan	√	MAGMA, JTI-PrediXcan	×	NA	0.359786028524236	0.15239712350494	0.125319051952641
TTC24	FUSION, JTI-PrediXcan	√	JTI-PrediXcan	×	NA	−0.112578051	0.0903664465752124	−0.036658648
ZBTB39	FUSION, JTI-PrediXcan	√	JTI-PrediXcan	×	NA	−0.023474569	−0.101597819	−0.185836871
B9D2	FUSION, MAGMA	×	NA	×	NA	0.365151042906335	−0.022523441	0.0693930801751631
CALCA	FUSION, MAGMA	×	NA	×	NA	2.36973513232638	0.0864374882312646	1.41224311053889
CSPG5	FUSION, MAGMA	×	NA	×	NA	1.04631306588356	0.586294556766031	0.147118031776585
DHX30	FUSION, MAGMA	×	NA	×	NA	0.224381852042872	0.159239728549263	−0.233115176
FHL5	FUSION, MAGMA	×	NA	√	FUSION, MAGMA	1.64468664258626	0.0327313463894807	0.376216783304532
HHIPL1	FUSION, MAGMA	×	NA	×	NA	0.314520642014664	−0.269973021	0.117091828856953
HJURP	FUSION, MAGMA	×	NA	×	NA	0.801295049083255	−0.177180613	−0.101596149
KTN1	FUSION, MAGMA	×	NA	×	NA	0.541569481289006	0.0696303706283601	−0.223006165
LRP1	FUSION, MAGMA	√	FUSION	√	FUSION, MAGMA, JTI-PrediXcan	3.67314339988961	0.134803950405592	0.803977371715713
MAP4	FUSION, MAGMA	×	NA	×	NA	0.845551776264265	−0.061472362	−0.041466686
NAB2	FUSION, MAGMA	×	NA	√	FUSION	1.20077407828595	−0.078670971	0.943103770824176
SFXN2	FUSION, MAGMA	×	NA	×	NA	0.420981586948363	0.082919704924982	0.392160387675544
SLC24A3	FUSION, MAGMA	×	NA	×	NA	0.837878120862327	0.100893017728755	0.665708322191937
SUGCT	FUSION, MAGMA	×	NA	×	NA	0.878587017841562	0.131659853992558	0.216142057032876
UFL1	FUSION, MAGMA	×	NA	√	FUSION, MAGMA, JTI-PrediXcan	0.137318524376632	−0.075747007	0.871789162394911
MRGPRE	FUSION, MAGMA, eQTL	×	NA	×	NA	0.623454352941175	0.105909178755406	0.193315828231861
STAT6	FUSION, MAGMA, JTI-PrediXcan	√	JTI-PrediXcan	√	FUSION, MAGMA, JTI-PrediXcan	3.00490427420246	0.707922348716691	2.29100240771294
CALCB	FUSION, MAGMA, mQTL	×	NA	×	NA	0.836950570707407	0.111723399679157	0.782220816061467
MEF2D	FUSION, MAGMA, mQTL	×	NA	√	FUSION, JTI-PrediXcan	1.8446832215119	0.103947682824509	1.24795266915079
PRDM16	FUSION, MAGMA, mQTL	×	NA	√	FUSION, MAGMA, mQTL	4.76496934758302	0.229525850990023	1.43549514400552
PHACTR1	FUSION, MAGMA, mQTL, JTI-PrediXcan	√	FUSION, JTI-PrediXcan	√	FUSION, JTI-PrediXcan	2.64863880801219	0.329033379461558	1.28491453226306
TMEM51	FUSION, mQTL	×	NA	×	NA	0.324723002719683	0.0175358582031461	−0.114450971
ALG12	JTI-PrediXcan	√	FUSION, MAGMA, JTI-PrediXcan	×	NA	−0.232168941	0.00221505422599431	−0.138432492
ANKK1	JTI-PrediXcan	√	FUSION, MAGMA, JTI-PrediXcan	×	NA	−0.009960156	−0.163077601	0.0184594591191178
ARIH2OS	JTI-PrediXcan	√	FUSION, JTI-PrediXcan	×	NA	0.290381289112369	0.0407333716497951	−0.012036367
ARL6IP4	JTI-PrediXcan	√	MAGMA, JTI-PrediXcan	×	NA	0.150787885327833	−0.041809937	0.227140200882244
C3orf62	JTI-PrediXcan	√	FUSION, JTI-PrediXcan	×	NA	0.200587778633348	0.146091349019112	0.00741614359066453
CCDC36	JTI-PrediXcan	√	MAGMA, JTI-PrediXcan	×	NA	NA	NA	NA
CCDC71	JTI-PrediXcan	√	FUSION, JTI-PrediXcan	×	NA	0.256585706910546	0.166309349327275	−0.078357276
CELSR3	JTI-PrediXcan	√	FUSION, MAGMA, eQTL, JTI-PrediXcan	×	NA	0.119999597557478	0.09429551597812	0.178045792245991
CRELD2	JTI-PrediXcan	√	FUSION, MAGMA, JTI-PrediXcan	×	NA	0.381953248424051	0.140496210618467	0.0676003643226458
DRC3	JTI-PrediXcan	√	FUSION, JTI-PrediXcan	×	NA	0.175710876496949	0.22497425544632	0.0145874102023276
HIST1H2BD	JTI-PrediXcan	√	MAGMA, JTI-PrediXcan	×	NA	NA	NA	NA
IP6K2	JTI-PrediXcan	√	FUSION, MAGMA, JTI-PrediXcan	×	NA	0.00707926381654044	0.325263806565343	−0.308951157
LINC02166	JTI-PrediXcan	√	FUSION, JTI-PrediXcan	×	NA	NA	NA	NA
MAEA	JTI-PrediXcan	√	FUSION, MAGMA, JTI-PrediXcan	×	NA	−0.296921421	0.991778979546547	0.00711687416957938
NCKIPSD	JTI-PrediXcan	√	MAGMA, JTI-PrediXcan	×	NA	0.170083332653109	0.163753771059098	−0.085890517
OGFOD2	JTI-PrediXcan	√	MAGMA, JTI-PrediXcan	×	NA	0.233177314686005	0.107483481869043	0.213635996087082
PIM3	JTI-PrediXcan	√	FUSION, JTI-PrediXcan	×	NA	0.275509582578445	−0.01763925	−0.249641656
RASGRF2	JTI-PrediXcan	√	FUSION, JTI-PrediXcan	×	NA	0.571025712599019	0.612050252046555	0.539191146561724
RERG	JTI-PrediXcan	√	FUSION, MAGMA, JTI-PrediXcan	×	NA	0.50096582432266	0.94236482303448	0.897008803529776
RPP21	JTI-PrediXcan	√	MAGMA, JTI-PrediXcan	×	NA	0.124184224357975	−0.023418647	−0.476777546
RRBP1	JTI-PrediXcan	√	MAGMA, JTI-PrediXcan	×	NA	−0.265711307	0.437368887395571	0.243605543130937
SLC26A6	JTI-PrediXcan	√	FUSION, MAGMA, eQTL, JTI-PrediXcan	×	NA	0.248037034603348	0.0326101601997732	−0.075148727
SMCR5	JTI-PrediXcan	√	FUSION, JTI-PrediXcan	×	NA	NA	NA	NA
SOX7	JTI-PrediXcan	√	MAGMA, JTI-PrediXcan	×	NA	−0.235742387	0.161934197469218	−0.171281885
SPATA33	JTI-PrediXcan	√	FUSION, JTI-PrediXcan	×	NA	−0.020486223	0.0474121846803396	−0.032791392
SREBF1	JTI-PrediXcan	√	FUSION, JTI-PrediXcan	×	NA	−0.047492627	0.198028017303947	−0.240782539
TMEM89	JTI-PrediXcan	√	FUSION, JTI-PrediXcan	×	NA	0.0973901895964615	−0.099338175	−0.03698422
TRIM10	JTI-PrediXcan	√	MAGMA, JTI-PrediXcan	×	NA	−0.12645781	−0.104819613	−0.103203709
TRIM26	JTI-PrediXcan	√	MAGMA, JTI-PrediXcan	×	NA	−0.151431133	0.0192352055323646	0.149795816481255
TRIM31	JTI-PrediXcan	√	MAGMA, JTI-PrediXcan	×	NA	0.0208054653841703	0.143075880887615	0.117138106328655
TRIM40	JTI-PrediXcan	√	MAGMA, JTI-PrediXcan	×	NA	−0.13590979	0.00663920590144585	−0.041325751
UQCRC1	JTI-PrediXcan	√	FUSION, JTI-PrediXcan	×	NA	0.322730330789477	0.132632615498281	−0.021382396
WDR6	JTI-PrediXcan	√	FUSION, JTI-PrediXcan	×	NA	−0.082409051	0.232064088655754	0.145731123558279
XKR6	JTI-PrediXcan	√	FUSION, JTI-PrediXcan	×	NA	0.218985261309357	0.121925434029205	0.0898575636645161
ZBED4	JTI-PrediXcan	√	FUSION, MAGMA, JTI-PrediXcan	×	NA	0.028836782724248	0.308754958148029	0.265081694171281
ZFAND5	JTI-PrediXcan	√	MAGMA, JTI-PrediXcan	×	NA	0.279721844161871	0.588195700698123	0.0947171576814631
ZNF697	JTI-PrediXcan	√	FUSION, JTI-PrediXcan	×	NA	−0.364594847	0.00442179768563452	0.142940889982599
ZNF808	JTI-PrediXcan	√	FUSION, JTI-PrediXcan	×	NA	0.0981477489738863	0.189390038746002	−0.057942593
REEP3	JTI-PrediXcan	√	JTI-PrediXcan	√	FUSION, JTI-PrediXcan	0.118243164040487	0.23020461305631	0.572021449697311
TJP2	MAGMA, eQTL	×	NA	×	NA	0.705879412025416	−0.629753827	0.0881011087319735
TRPM8	MAGMA, mQTL	×	NA	×	NA	0.657785153855803	−0.057995162	0.502279618117342
CACNA1A	NA	NA	FUSION, MAGMA	×	NA	0.753372975465257	2.53671237848025	−0.255234053
KLHDC8B	NA	NA	FUSION, MAGMA	×	NA	0.404411695783461	0.335351528678147	0.0987640739918263
ACO2	NA	NA	NA	NA	FUSION, JTI-PrediXcan	0.0888046564808445	0.0269877764599751	0.465084717303068
BCAR1	NA	NA	NA	NA	FUSION, JTI-PrediXcan	−0.036898536	−0.210686811	−0.012173616
CCDC134	NA	NA	NA	NA	FUSION, JTI-PrediXcan	0.0741073757871275	0.075451494580366	0.212591586718194
CFDP1	NA	NA	NA	NA	FUSION, MAGMA, JTI-PrediXcan	0.325398088367892	0.13320628367269	0.416450599434954
CHADL	NA	NA	NA	NA	FUSION, JTI-PrediXcan	0.18369554796958	0.167523392886105	0.165344455281645
GLCE	NA	NA	NA	NA	FUSION, JTI-PrediXcan	0.262511443361146	−0.113172068	0.551782310544156
ITPKB	NA	NA	NA	NA	FUSION, MAGMA, JTI-PrediXcan	0.349148413502103	−0.225519525	1.31939005847472
L3MBTL2	NA	NA	NA	NA	FUSION, JTI-PrediXcan	0.287926091959072	0.486445143650934	0.251500321836007
MED19	NA	NA	NA	NA	FUSION, JTI-PrediXcan	0.0353095246676063	−0.409493228	−0.008130699
PAM	NA	NA	NA	NA	FUSION, JTI-PrediXcan	−0.481055165	0.603875519769939	1.14298651236614
PPP2R5A	NA	NA	NA	NA	FUSION, JTI-PrediXcan	−0.085714562	0.165926735029786	0.217291415404394
RHOF	NA	NA	NA	NA	FUSION, JTI-PrediXcan	0.148481422282098	−0.14003072	0.507150954767214
SREBF2	NA	NA	NA	NA	FUSION, JTI-PrediXcan	−0.160664588	0.426017810166225	0.435117715897074
TEF	NA	NA	NA	NA	FUSION, JTI-PrediXcan	0.217670506634366	0.234955018252937	0.236393961733179
TMEM170A	NA	NA	NA	NA	FUSION, JTI-PrediXcan	0.00148289361765374	0.136070609755002	0.120312782524287
TNFRSF13C	NA	NA	NA	NA	FUSION, JTI-PrediXcan	−0.454208941	−0.383619352	−0.128668956
ZC3H7B	NA	NA	NA	NA	FUSION, MAGMA, JTI-PrediXcan	0.137018599759484	−0.050569323	0.00247931245476753

Column headers: Gene, Positive genes identified. Methods (Trait), Analytical approaches used for each trait-specific analysis (Overall Migraine, Migraine with Aura [*MA*], Migraine without Aura [*MO*]) MA/MO, Presence (√) or absence (*NA*) of the gene in the MA or MO subtype lists derived from the Overall Migraine reference panel. A checkmark (√) indicates that the MA/MO-specific gene overlaps with the Overall Migraine-positive gene list, while NA denotes no overlap. PoPS Score (*Trait*), Gene prioritization score from the PoPS framework for each trait (Overall Migraine, MA, MO).

Notes: Reference panel, The Overall Migraine-positive gene list serves as the primary reference. Genes marked with “√” in the MA/MO columns are shared with the Overall Migraine list, while NA indicates subtype-specific genes absent in OM. Methods annotation: If a trait-specific Methods column (e.g., “Methods (Overall Migraine)”) is labeled NA, it indicates that the gene was identified in MA/MO but not in Overall Migraine. Inclusion criterion: All listed genes were significant in at least two analytical methods for at least one trait. Symbols:√: Gene overlaps between the subtype (MA/MO) and Overall Migraine. NA: No overlap between the subtype (MA/MO) and Overall Migraine. Example interpretation: For PHACTR1 (Methods (Overall Migraine), *FUSION*, *MAGMA*, *mQTL*, *JTI-PrediXcan,* *MA,* √, *MO,* √), the checkmarks confirm its presence in both MA and MO lists derived from Overall Migraine, with high PoPS Scores across all traits (Overall Migraine: 2.65, *MA*: 0.33, *MO*: 1.28). For RERG (Methods (Overall Migraine), *NA,* *MA,* √, *MO,* *NA*), the NA in “Methods (Overall Migraine)” indicates it was identified in MA but not in Overall Migraine. This table highlights genes with cross-subtype consistency or subtype specificity, prioritized by methodological robustness and phenotypic relevance. *FUSION *Functional Summary-based Imputation, *MAGMA *Multi-marker Analysis of GenoMic Annotation, *mQTL *Methylation Quantitative Trait Loci, *eQTL* Expression Quantitative Trait Loci, *JTI-PrediXcan* Joint-Tissue Imputation Enhanced PrediXcan, *PoPS* Polygenic Priority Score

**Table 3:** Statistical validation of tissue-specific MA and MO enrichment across spatially resolved murine organ regions using the GsMap algorithm with cauchy combination tests

annotation	*p*_cauchy_(MA)	*p*_median_(MA)	*p*_cauchy_(MO)	*p*_median_(MO)
Cartilage	0.001989966	0.038852604	0.000381139	0.050527718
Jaw and tooth	0.003904829	0.141825932	0.000583311	0.016264954
Cartilage primordium	0.012272543	0.122737728	0.000664256	0.021000919
Connective tissue	0.024432631	0.096181151	0.002125124	0.038464403
Mucosal epithelium	0.027639135	0.172755272	0.00232748	0.031748097
Lung	0.031452614	0.332030211	0.003264737	0.026035984
Meninges	0.032544189	0.090544602	0.005124826	0.079225257
Kidney	0.033560443	0.177016842	0.006535275	0.031672725
GI tract	0.03598681	0.134574184	0.010118752	0.082811706
Inner ear	0.049208145	0.195626826	0.010658896	0.032342653
Submandibular gland	0.060975461	0.159003375	0.011595545	0.106249565
Adipose tissue	0.06386762	0.139983882	0.013616844	0.073440821
Muscle	0.088866265	0.204478963	0.019531163	0.087727565
Adrenal gland	0.093269614	0.234082467	0.035138463	0.119015463
Choroid plexus	0.095712793	0.287723657	0.044570075	0.100558032
Smooth muscle	0.107115547	0.240173689	0.045962044	0.138612758
Brain	0.115143314	0.221362095	0.068530434	0.222520925
Epidermis	0.132507572	0.323154373	0.087729798	0.272284051
Sympathetic nerve	0.14419019	0.31973193	0.128177824	0.188094815
Dorsal root ganglion	0.149228734	0.329010516	0.226310633	0.272710625
Spinal cord	0.160259216	0.312185247	0.235648717	0.276595869
Liver	0.215698278	0.285288797	0.296550361	0.415234415
Heart	0.224998398	0.348309827	0.418357735	0.46242447
Bone	0.313597694	0.358625962	0.996361999	0.586744823
Cavity	0.741609388	0.472535142	0.997954333	0.292827

The annotation denotes 25 embryonic mouse organs, p_cauchy_ represents the P-value from the Cauchy combination test, and p_median_ indicates the P-value derived from the median-based validation test

*MA* Migraine with aura, *MO* Migraine without aura, *gsMap* Genetically informed spatial mapping of cells for complex traits

### Tissue enrichment specificity of MA and MO in sc-ST

**Fig. 5:**
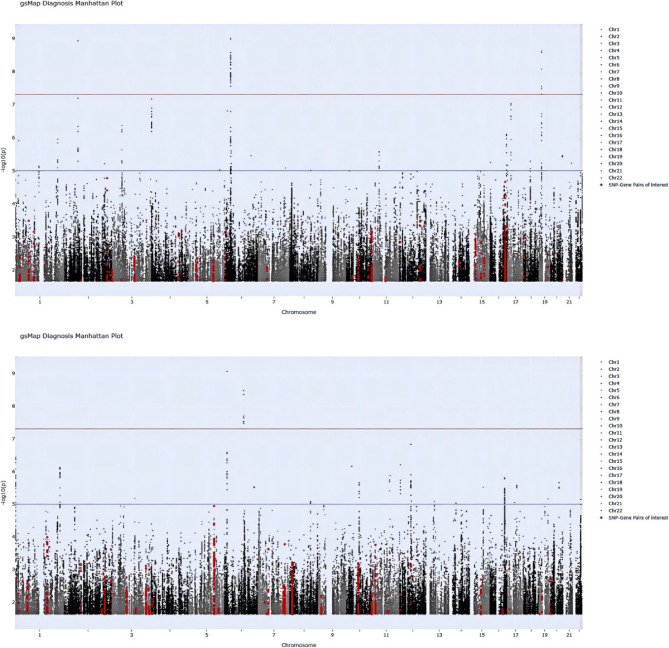
Genome-wide spatial association signals of MA and MO-associated genes identified by gsMap. From top to bottom, the Manhattan plots of MA and MO, respectively. Chromosomal positions (x-axis) and -log10p values (y-axis) are shown. MA: migraine with aura; MO: migraine without aura; gsMap: genetically informed spatial mapping of cells for complex traits

**Fig. 6:**
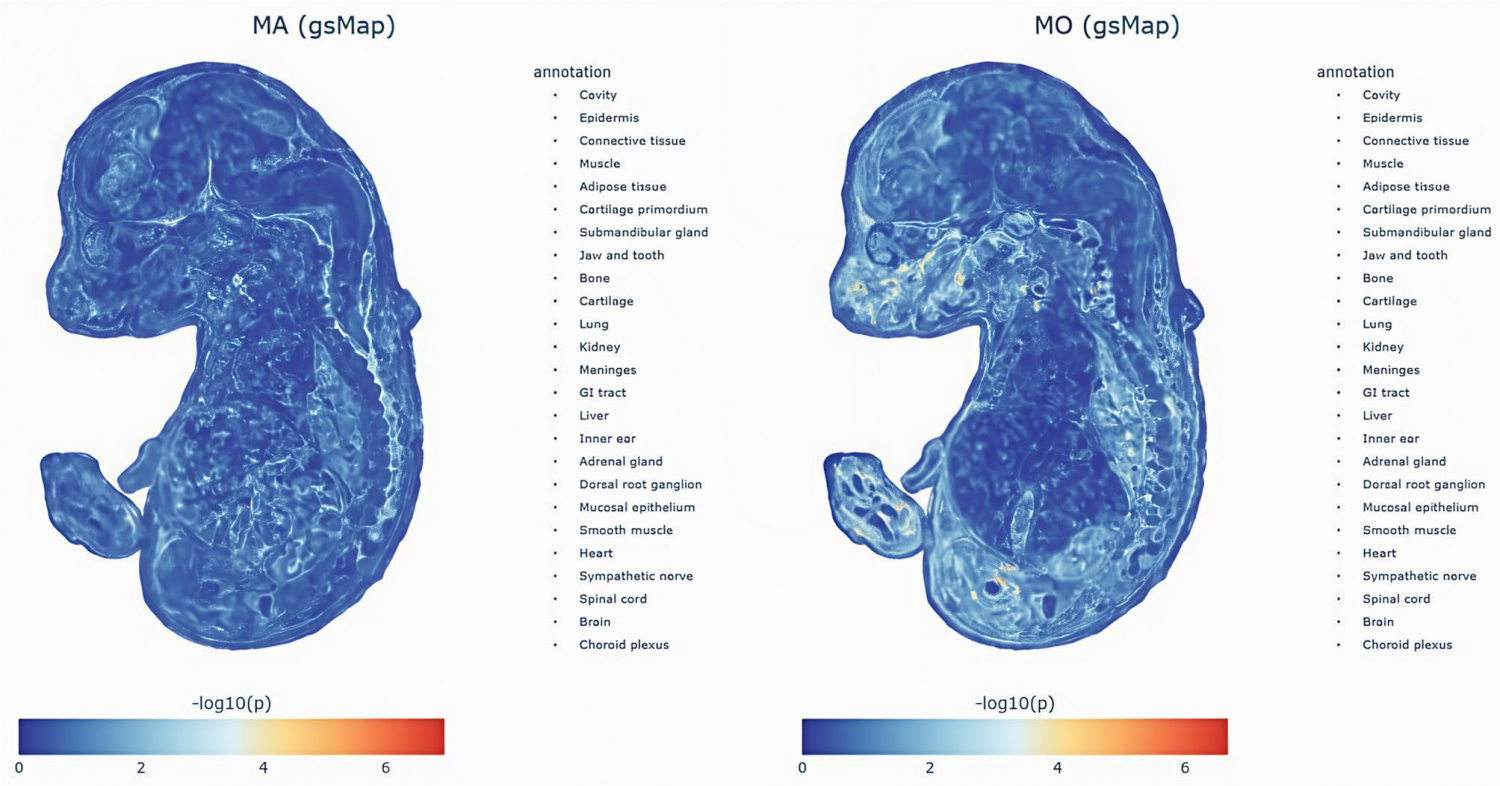
Spatial mapping of MA and MO-associated cellular patterns in E16.5 mouse embryonic single-cell spatial transcriptomics (ST) data, generated by gsMap algorithm across 25 organs. MA: migraine with aura; MO: migraine without aura; gsMap: genetically informed spatial mapping of cells for complex traits

 Fig. 5 displays genome-wide spatial association signals of MA- and MO-associated genes identified by the gsMap algorithm. The gsMap-derived gene-specific scores (GSS) are cataloged in Supplementary Tables S14-S15, with spatially resolved LDSC enrichment of tissue-specific MA and MO across murine organ regions detailed in Supplementary Tables S16-S17. Spatial organ mapping results (Supplementary Tables S18-S19) and their integrative visualization (Fig. 6) further delineate MetS-associated cellular patterns in E16.5 mouse embryos at single-cell resolution. Statistical validation using the Cauchy combination test (Table 2) identified 25 organs/tissues showing significant enrichment associations. Single-cell spatial transcriptomic analysis revealed distinct enrichment patterns between MA and MO across E16.5 murine embryonic organs, demonstrating complementary associations with genetic enrichment profiles from LDSC-SEG and MAGMA analyses. MO exhibited a pronounced peripheral tissue tropism in spatial mapping, with significant associations in cartilage (MO: p_Cauchy_ = 0.00038 vs. MA: p_Cauchy_ = 0.00199), jaw/tooth (MO: p_Cauchy_ = 0.00058 vs. MA: p_Cauchy_ = 0.0039), and meninges (MO: p_Cauchy_ = 0.0051). These findings align with LDSC-SEG and MAGMA results, which consistently prioritized vascular tissues (e.g., tibial artery) and gastrointestinal systems (transverse colon, gastroesophageal junction), suggesting MO pathogenesis may center on peripheral vascular dysfunction and mucosal barrier disruption. In contrast, while MA partially overlapped with MO in cartilage primordium (p_Cauchy_ = 0.012) and mucosal epithelium (p_Cauchy_ = 0.0276), its weaker meningeal association (p_Cauchy_ = 0.0325) and adrenal divergence (MA: p_Cauchy_ = 0.093 vs. MO: p_Cauchy_ = 0.035) contrasted with MAGMA-implicated neuroimmune mechanisms (basal ganglia, hypothalamus) and the absence of LDSC-SEG signals. This discrepancy implies MA’s neuroregulatory abnormalities may involve non-heritable pathways, such as epigenetic modifications or cell-cell interactions. Notably, neither subtype reached significance in brain tissue (MA: *p* = 0.115; MO: *p* = 0.0685), though MO’s lower p-value synergized with MAGMA’s vascular tissue signals, reinforcing its peripherally-driven hypothesis. Integrating spatial transcriptomic and genetic enrichment analyses, we propose MO mechanisms converge on heritable peripheral tissue dysregulation, whereas MA likely involves dynamic modulation of central neuroimmune microenvironments.

## Discussion

This study systematically dissects the genetic heterogeneity and molecular mechanisms underlying migraine and its subtypes (MA and MO) through an integrative framework combining large-scale GWAS, multi-omics functional annotations, cross-method validation, and sc-ST. Leveraging subtype-stratified data from FinnGen R11 and global GWAS repositories, we confirmed robust genetic correlations between MA and MO while revealing subtype-specific divergence in functional genomic annotations via partitioned heritability (S-LDSC). Crucially, sc-ST mapping of E16.5 murine embryos (25 organs) resolved spatially enriched patterns that complemented and extended genetic findings: MO exhibited pronounced peripheral tropism in vascular tissues and mucosal epithelia, aligning with LDSC-SEG/MAGMA-prioritized pathways (e.g., ITPKB, CFDP1), whereas MA showed preferential enrichment in hypothalamic nuclei and microglia-adjacent regions, corroborating its neuroimmune etiology (CACNA1A, STAT6). By integrating sc-ST with multi-omics profiling (TWAS, SMR, PoPS), we uncovered microenvironment-specific co-localization of cross-subtype core genes (e.g., PHACTR1-STAT6 clusters in meningeal vascular niches) and developmental origins of subtype divergence, such as prenatal enrichment of migraine-associated loci in neural crest-derived tissues (e.g., jaw primordium). The methodological innovation of combining FUSION, MAGMA, JTI-PrediXcan, and sc-ST not only enhanced gene prioritization robustness but also bridged spatial-genetic hierarchies, while the expanded data ecosystem—spanning 53 tissue transcriptomes, methylation QTLs, and embryo-wide spatial atlases—provided unprecedented resolution into neurovascular coupling, neuroinflammation, and epigenetic regulation. For clinicians, these insights translate into three actionable priorities: [1] nuanced patient counseling emphasizing MA’s neuroimmune aura mechanisms versus MO’s vascular-peripheral pain pathways; [2] subtype-guided therapeutic selection (e.g., neuronal excitability modulators for MA, vascular/CGRP-targeted agents for MO); and [3] early adoption of emerging biologics targeting prioritized genes like STAT6 (MA) or TRPM8 (MO) as clinical evidence evolves.

The distinct tissue enrichment patterns of MA and MO, revealed through integrated analyses of LDSC-SEG, MAGMA, and sc-ST, underscore their divergent pathophysiological mechanisms. This complements the comprehensive framework presented by Raggi et al. [36] in their hallmark review of migraine pathophysiology, which systematically catalogues how such molecular pathways translate to clinical phenotypes. While MA exhibited potential associations with central nervous and immune microenvironments in sc-ST mapping, these signals lacked statistical significance in LDSC-SEG, contrasting with MAGMA’s tentative links to basal ganglia and hypothalamic regulation [37], implicating neuronal hyperexcitability and cortical spreading depression [38, 39]. MAGMA and sc-ST also revealed weak associations with cultured fibroblasts in MA, possibly indicating indirect roles of glial or peripheral immune cells [40–42]. However, LDSC-SEG did not support these signals, suggesting MA’s neuroimmune mechanisms may involve epigenetic or post-transcriptional regulation rather than genetic enrichment alone. Conversely, MO showed consistent enrichment in peripheral vascular tissues and gastrointestinal regions. These findings suggest MO pathology involves vascular smooth muscle dysfunction (e.g., TRPM8-mediated cold sensitivity [43–45]) and gut-brain axis interactions (e.g., CGRP signaling via CALCA [46, 47]). Petschner et al. [48] provide additional clinical context, demonstrating that MO patients exhibit downregulated retinoic acid signaling (via CYP26B1) and immune-metabolic pathways in blood transcriptomics, which may explain the comorbidity between MO and gastrointestinal disorders. Their identification of LRP1 polymorphisms as regulators of retinoic acid availability offers a mechanistic link to our observed vascular dysregulation in MO, suggesting dietary or supplemental retinoic acid modulation as a potential adjunct therapy. Genetically, MA overlaps with epilepsy-related loci (e.g., PRRT2 [49, 50]), while MO is enriched in pain-modulatory genes (e.g., LRP1) and CGRP pathways (CALCA) [47, 51].

Our integrative multi-omics analysis systematically reveals significant genetic heterogeneity between migraine subtypes. The low-density lipoprotein receptor gene LRP1 plays a central role in maintaining blood-brain barrier integrity while also regulating neuronal calcium signaling and synaptic plasticity in MA [52]. Recent studies demonstrate that LRP1 polymorphisms significantly influence the propagation and duration of cortical spreading depression (CSD), potentially explaining the visual aura in MA patients [53]. In MO, LRP1 primarily modulates peripheral vascular tone and pain signaling through calcium channel regulation in vascular smooth muscle cells. The dual role of LRP1 in both subtypes, as highlighted by Sun et al. [54] and Petschner et al. [48], underscores its potential as a pleiotropic target for migraine therapy, particularly given its involvement in oxidative stress (MO) and retinoic acid metabolism (MA-MO overlap). The vascular regulator PHACTR1 shows strong associations with cerebrovascular abnormalities (e.g., arterial dissection) in MA [55]. GWAS data indicate that the PHACTR1 rs9349379 polymorphism not only increases MA risk but also correlates with post-stroke migraine [56]. In MO, PHACTR1 appears to influence headache initiation through peripheral vascular contraction-dilation balance. The STAT6 gene provides novel insights into migraine’s neuroimmune mechanisms. In MA, STAT6 promotes neurogenic inflammation and blood-brain barrier disruption via mast cell activation [57], while STAT6-deficient mice show attenuated CSD responses [47]. In MO, STAT6 mediates peripheral nociceptor sensitization through IL-4/IL-13 signaling in macrophages. Although methodological support varies across subtypes, REEP3 plays important roles in neuronal plasticity. Transcriptomic analysis reveals upregulated REEP3 expression in MA prefrontal cortex, potentially contributing to aura through glutamatergic signaling [37]. In MO, REEP3 associates with peripheral nociceptor sensitization [58]. Among MO-specific genes, ITPKB enhances trigeminovascular sensitivity by regulating calcium release in nociceptive neurons [58]. Single-cell sequencing shows ITPKB’s specific expression in small-diameter trigeminal ganglion neurons, potentially explaining MO patients’ mechanical and thermal hypersensitivity. The transcriptional regulator FHL5 interacts with voltage-gated calcium channels (e.g., CACNA1A) to modulate neuronal excitability in MA [59], while influencing pain-related gene expression through histone deacetylation in MO [60]. Additional genes contribute through diverse mechanisms: MEF2D regulates synaptic plasticity and pain pathways [61] while NAB2 modulates NGF signaling in nociceptive neurons [52]. Our novel findings also implicate UFL1 and ZBTB39 in migraine chronification - UFL1 stabilizes inflammatory proteins [37], and ZBTB39 epigenetically modifies pain-related genes [60]. The distinct molecular pathways underlying MA and MO pathogenesis highlight the need for subtype-specific treatment approaches. Most critically, our integrated framework bridges the “genotype-phenotype gap” that has long hindered migraine management. By systematically identifying and validating subtype-specific molecular networks, we directly address the unmet need for refractory migraine biomarkers highlighted in Raggi et al.‘s [36] comprehensive review.

Our study has several limitations requiring consideration. First, GWAS was conducted exclusively in European ancestry populations, potentially limiting the generalizability of findings to other geographic cohorts. Second, TWAS relied on post-mortem expression profiles from GTEx v8, introducing potential biases due to tissue-specific degradation or confounding factors unrelated to in vivo metabolic states. Third, the PoPS framework excluded genes with incomplete functional annotations, introducing selection bias in candidate prioritization. Finally, while our multi-omics framework revealed divergent molecular architectures between migraine subtypes (MA: neuroimmune-epigenetic dysregulation; MO: vascular-metabolic perturbations), the mechanistic roles of the identified genes require rigorous functional validation to establish them as drivers of clinically distinct disease. This necessitates integrated approaches—including single-cell sequencing, animal models, and multi-ethnic replication—to inform clinical translation. Critically, although prior clinical studies suggest differential treatment responses (e.g., triptan efficacy), our study did not directly correlate the identified genetic and spatial signatures with therapeutic outcomes. Therefore, future work integrating subtype-stratified clinical trial data or experimental models (e.g., MA/MO patient-derived cellular systems) is essential to bridge this gap and confirm whether the proposed pathomechanisms fully account for the observed phenotypic heterogeneity. These limitations underscore the need for broader population sampling and functional genomics to refine precision strategies for migraine management.

## Conclusion

This study delineates the genetic and spatial architecture of migraine subtype (MA/MO) divergence through integrative multi-omics, combining cross-validated genetic analyses (LDSC-SEG, MAGMA) with sc-ST. Despite shared genetic correlations, sc-ST identified distinct spatial pathophysiological niches: MA showed prenatal enrichment in neural crest-derived tissues and hypothalamic microglial niches, implicating neuroimmune/epigenetic regulation. Conversely, MO exhibited peripheral tropism within vascular smooth muscle and gut-brain axis interfaces, validated by pathway analyses. Multi-modal frameworks prioritized core genes and subtype-exclusive loci, highlighting divergent neurovascular, inflammatory, and epigenetic mechanisms. Resolving spatial-genetic hierarchies via sc-ST uncovered developmental origins of divergence and microenvironmental drivers invisible to bulk analyses. These findings provide a nuanced pathophysiological understanding to inform patient counseling, guide subtype-specific therapeutic strategies, and support adoption of emerging targeted therapies. Critically, our results reconcile key clinical insights: [1] Oxidative stress and neurovascular coupling bridge MA/MO mechanisms; [2] The gut-brain axis critically modulates peripheral nociception in MO; [3] Stratified management is imperative for refractory cases, especially MA with central neuroimmune dysregulation. By integrating spatial-genetic hierarchies with clinical heterogeneity, this work establishes a roadmap for next-generation migraine therapeutics.

## Supplementary Information

Supplementary Material 1.

Supplementary Material 2.

Supplementary Material 3.

Supplementary Material 4.

Supplementary Material 5.

Supplementary Material 6.

Supplementary Material 7.

Supplementary Material 8.

Supplementary Material 9.

## Data Availability

The R packages and Python-based software used in this study, along with their links and descriptions, are detailed in Supplementary Table S2. All SMR results and GWAS/QTL associations for the selected SNPs are provided in Supplementary Tables. It is important to note that the data used for MR analysis are not raw. Instead, they represent newly generated meta-analyzed data by combining raw data from multiple high-quality studies for each trait. These data have undergone heterogeneity testing and correction for sample overlap. The original datasets and their links are provided in Supplementary Table S1. The newly generated meta-analyzed data are not publicly available; interested researchers should contact the corresponding author for access. We encourage interested readers to obtain data from their respective sources.

## Abbreviations

ACO2: Aconitase 2
ATP: Adenosine triphosphate
BCAR1: Breast cancer anti-estrogen resistance 1
BSGS: Brisbane systems genetics study
CACNA1A: Calcium voltage-gated channel subunit alpha1 A
CCDC: Coiled-coil domain containing
CELF3: CUGBP elav-like family member 3
CELSR3: Cadherin EGF LAG seven-pass G-Type receptor 3
CFDP1: Craniofacial development protein 1
CHADL: Chondroadherin like
CGRP: Calcitonin gene-related peptide
CSD: Cortical spreading depression
eQTL: expression Quantitative trait loci
ENCODE: Encyclopedia of DNA elements
FDR: False discovery rate
FHL5: Four and a half LIM domains 5
FUSION: Functional summary-based imputation
GI: Gastrointestinal
GLCE: Glucuronyl C5-epimerase
gnomAD: Genome aggregation database
GO: Gene ontology
gsMap: Genetically informed spatial mapping of cells for complex traits
GTEx: Genotype-tissue expression project
GWAS: Genome-wide association study
HDL: High-definition likelihood
HEIDI: Heterogeneity in dependent instruments test
ICHD-3: International classification of headache disorders, 3rd edition
IP6K2: Inositol hexakisphosphate kinase 2
ITPKB: Inositol-trisphosphate 3-kinase B
JTI-PrediXcan: Joint-tissue imputation enhanced prediXcan analysis
KEGG: Kyoto encyclopedia of genes and genomes
KLHDC8B: Kelch domain containing 8B
L3MBTL2: L3MBTL histone methyl-lysine binding protein 2
LBC: Lothian birth cohort
LDSC: Linkage disequilibrium score regression
LDSC-SEG: Linkage disequilibrium score regression-stratified by expression of genes
LRP1: LDL receptor related protein 1
MA: Migraine with aura
MAGMA: Multi-marker analysis of genoMic annotation
MAF: Minor allele frequency
MD: Doctor of medicine
MED19: Mediator complex subunit 19
MEF2D: Myocyte enhancer factor 2D
MO: Migraine without aura
mQTL: methylation Quantitative trait loci
NAB2: NGFI-A binding protein 2
NCKIPSD: NCK Interacting protein with SH3 domain
NSAIDs: Non-steroidal anti-inflammatory drugs
OGFOD2: 2-Oxoglutarate and iron dependent oxygenase domain containing 2
PAM: Peptidylglycine alpha-amidating monooxygenase
PhD: Doctor of philosophy
PHACTR1: Phosphatase and actin regulator 1
pLI: Probability of loss-of-function intolerance
PoPS: Polygenic priority score
PPP2R5A: Protein phosphatase 2 regulatory subunit b’alpha
PRDM16: PR/SET Domain 16
Reactome: Reactome pathway database
REEP3: Receptor accessory protein 3
REML: Restricted maximum likelihood
RERG: RAS Like estrogen regulated growth inhibitor
RHOF: Ras Homolog family member f, filopodia associated
r_g_: genetic correlation
RRBP1: Ribosome binding protein 1
sc-ST: single-cell spatial transcriptomics
SE: Standard error
S-LDSC: Sparse linkage disequilibrium score regression
SLC26A6: Solute carrier family 26 member 6
SMR: Summary mendelian randomization
SNP: Single nucleotide polymorphism
STAT6: Signal transducer and activator of transcription 6
STRING: Search tool for the retrieval of interacting genes/proteins
SREBF2: Sterol regulatory element binding transcription factor 2
TEF: Thyrotrophic embryonic factor
TMEM170A: Transmembrane Protein 170 A
TNFRSF13C: TNF Receptor superfamily member 13 c
TRIM: Tripartite motif containing
TRPM8: Transient receptor potential cation channel subfamily m member 8
TTC24: Tetratricopeptide repeat domain 24
TWAS: Transcriptome-wide association study
UFL1: UFM1 specific ligase 1
UTMOST: Unified test for molecular signature
ZBTB39: Zinc finger and btb domain containing 39
ZC3H7B: Zinc finger ccch-type containing 7b
ZFAND5: Zinc finger an1-type containing 5
ZNF: Zinc finger protein
