# Supplementary material for: Unveiling migraine subtype heterogeneity and risk loci: integrated genome-wide association study and single-cell transcriptomics discovery
Source: J Headache Pain. 2025 Aug 18;26(1):185. doi: 10.1186/s10194-025-02128-7 (PMC12360011; doi:10.1186/s10194-025-02128-7)
Supplement: Supplementary file 1 — Supplementary Material 1. [file 10194_2025_2128_MOESM1_ESM.docx]

### ****General Abbreviations:****

**ADAMTSL4**: ADAMTS Like 4
**AMPP**: American Migraine Prevalence and Prevention
**ATP**: Adenosine Triphosphate
**BBB**: Blood-Brain Barrier
**CGRP**: Calcitonin Gene-Related Peptide
**CSD**: Cortical Spreading Depression
**eQTL**: Expression Quantitative Trait Loci
**FDR**: False Discovery Rate
**GWAS**: Genome-Wide Association Study
**HDL**: High-Definition Likelihood
**HEIDI**: Heterogeneity in Dependent Instruments Test
**ICHD-3**: International Classification of Headache Disorders, 3rd edition
**LD**: Linkage Disequilibrium
**LDSC**: Linkage Disequilibrium Score Regression
**MA**: Migraine with Aura
**MAF**: Minor Allele Frequency
**MO**: Migraine without Aura
**mQTL**: Methylation Quantitative Trait Loci
**NSAIDs**: Non-Steroidal Anti-Inflammatory Drugs
**PoPS**: Polygenic Priority Score
**REML**: Restricted Maximum Likelihood
**S-LDSC**: Sparse Linkage Disequilibrium Score Regression
**SMR**: Summary Mendelian Randomization
**SNP**: Single Nucleotide Polymorphism
**TWAS**: Transcriptome-Wide Association Study

### ****Gene/Protein Abbreviations:****

**CACNA1A**: Calcium Voltage-Gated Channel Subunit Alpha1 A
**CALCA**: Calcitonin Gene-Related Peptide Alpha
**CFDP1**: Craniofacial Development Protein 1
**FHL5**: Four and a Half LIM Domains 5
**ITPKB**: Inositol-Trisphosphate 3-Kinase B
**KLHDC8B**: Kelch Domain Containing 8B
**LRP1**: Low-Density Lipoprotein Receptor-Related Protein 1
**PHACTR1**: Phosphatase and Actin Regulator 1
**PRDM16**: PR Domain Containing 16
**RASGRF2**: Ras Protein-Specific Guanine Nucleotide-Releasing Factor 2
**REEP3**: Receptor Accessory Protein 3
**STAT6**: Signal Transducer and Activator of Transcription 6
**TRPM8**: Transient Receptor Potential Cation Channel Subfamily M Member 8

### ****Datasets & Consortia:****

**BSGS**: Brisbane Systems Genetics Study
**eQTLGen**: Expression Quantitative Trait Loci Consortium
**FinnGen R11**: Finnish Biobank Study Release 11
**GTEx**: Genotype-Tissue Expression Project
**LBC**: Lothian Birth Cohort

### ****Methods & Tools:****

**FUSION**: Functional Summary-based Imputation
**JTI-PrediXcan**: Joint-Tissue Imputation Enhanced PrediXcan
**MAGMA**: Multi-marker Analysis of GenoMic Annotation
**LDSC-SEG**: Linkage Disequilibrium Score Regression-Stratified by Expression of Genes
